# Associations between redox-related biomarkers and treatment response in patients with inflammatory bowel disease initiating infliximab or vedolizumab

**DOI:** 10.3389/fphar.2026.1908844

**Published:** 2026-10-14

**Authors:** Sem Geertsema, Roberta Loveikyte, Sofie de Jong, Marian L. C. Bulthuis, Marjan Reinders-Luinge, Harry van Goor, Klaas Nico Faber, Arno R. Bourgonje, Gerard Dijkstra

**Affiliations:** 1 Department of Gastroenterology and Hepatology, University of Groningen, University Medical Center Groningen, Groningen, Netherlands; 2 Department of Gastroenterology and Hepatology, Leiden University Medical Center, Leiden, Netherlands; 3 Department of Pathology and Medical Biology, University of Groningen, University Medical Center Groningen, Groningen, Netherlands; 4 The Dr. Henry D. Janowitz Division of Gastroenterology, Department of Medicine, Icahn School of Medicine at Mount Sinai, New York, NY, United States

**Keywords:** biologic therapy, biomarkers, exploratory research, inflammatory bowel disease, iron metabolism, oxidative stress, redox

## Abstract

**Background:**

Oxidative stress is increasingly recognized as a key component of inflammatory bowel disease (IBD) pathophysiology, yet the clinical relevance of systemic redox biomarkers during biologic therapy remains poorly understood. In this exploratory study, we investigated whether systemic redox biomarkers are associated with short-term and long-term response to infliximab (IFX) or vedolizumab (VEDO) and their relationship with iron metabolism.

**Materials and Methods:**

Patients with active IBD initiating induction therapy with IFX or VEDO were included. Serum redox biomarkers (cysteine, homocysteine, free thiols (FTs), glutathione, and thioredoxin-1), inflammatory markers, and iron parameters were measured at baseline and during induction therapy. Patients were stratified according to short-term clinical response at week 14 and long-term response at 1 year. Longitudinal mixed-effects models, correlation analyses, and logistic regression were used to evaluate associations between redox biomarkers, treatment outcomes, and iron metabolism.

**Results:**

Fifty-eight patients were included (IFX: n = 37; VEDO: n = 21). Induction therapy resulted in marked reductions in inflammatory markers in short-term responders. Redox biomarkers showed more modest, mostly non-significant, response-dependent patterns. IFX-treated short-term non-responders exhibited non-significant lower cysteine levels than responders at baseline (2,336 vs. 3,611 μg/mL; p > 0.05) and week 14 (2,888 vs. 3,599 μg/mL; p > 0.05). Similarly, homocysteine levels were higher in IFX-treated short-term non-responders at baseline (7,500 vs. 4,260 ng/mL; p < 0.05) and week 14 (7,002 vs. 4,278 ng/mL; p > 0.05), whereas VEDO-treated patients showed comparable levels between response groups. FT levels showed no significant changes, increasing in short-term responders while decreasing in short-term non-responders across both therapies. Glutathione levels showed no major overall changes, although levels between IFX-treated long-term responders and non-responders appeared to differ during induction therapy. Glutathione correlated inversely with transferrin saturation (r = −0.50, p < 0.05) and serum iron (r = −0.46, p < 0.05) after induction therapy.

**Conclusion:**

Exploratory analyses suggested treatment response-related differences in cysteine, homocysteine, and FTs in IFX-treated patients, highlighting their potential as biomarkers of treatment response that warrant further investigation. Glutathione demonstrated an association with iron metabolism, suggesting a different biological role. These findings suggest a potential role for redox-iron interactions in treatment response biology that warrant further exploration and validation in larger prospective cohorts.

## 1 Introduction

Inflammatory bowel diseases (IBD), encompassing Crohn’s disease (CD) and ulcerative colitis (UC), are chronic immune-mediated diseases marked by recurrent episodes of intestinal inflammation (Abraham and Cho, 2009). The relapsing nature of IBD complicates disease monitoring and emphasizes the need for reliable and minimally invasive biomarkers, allowing timely detection of inflammatory activity. Although endoscopy remains the diagnostic standard, its suitability for serial monitoring and assessment of treatment response is limited by the invasiveness and associated costs (Abraham and Cho, 2009; Church, 2013). Fecal calprotectin is currently the best noninvasive biomarker of mucosal inflammation, yet its performance is suboptimal for grading disease severity or guiding early therapeutic decisions (Ricciuto and Griffiths, 2019). These limitations have driven interest in blood-based biomarkers that can more accurately reflect intestinal inflammation and predict treatment response.

Oxidative stress, defined as an imbalance between oxidants and antioxidants favoring the oxidants, plays an important pathogenic role in IBD (Sies and Jones, 2020; Sies, 2015; Quattrini et al., 2026). Intestinal inflammation is accompanied by increased production of reactive oxygen species (ROS), contributing to epithelial damage, impaired barrier function, and chronic immune activation. This inflammatory environment is mirrored systemically: patients with IBD consistently exhibit signs of altered circulating redox homeostasis (Bourgonje et al., 2019; Bourgonje et al., 2020). Within the extracellular redox environment, free thiols (FTs) serve as readily oxidizable antioxidants that decrease during oxidative stress (Bourgonje et al., 2019; Bohra et al., 2025). Accordingly, high circulating FT levels reflect a more reduced extracellular redox state and therefore lower oxidative stress, whereas depletion of FTs indicates increased oxidative stress. Low-molecular-weight thiols, including cysteine, glutathione, and homocysteine, participate in redox buffering, transsulfuration, and cellular antioxidant defense (Cortese-Krott et al., 2017; Chiang et al., 2022; Perna et al., 2003; Jakubowski and Witucki, 2025). Thioredoxin-1 (Trx1), a ubiquitous disulfide-reducing protein, is upregulated in response to oxidative and inflammatory stimuli and has previously been linked to IBD activity (Zhou et al., 2010). These redox markers have been associated with clinical disease severity and inflammatory biomarkers (Bourgonje et al., 2019; Bohra et al., 2025; Geertsema et al., 2026). Importantly, redox biomarkers may provide complementary information by capturing aspects of immune activation and tissue injury that are not fully reflected by conventional inflammatory indices, such as C-reactive protein or fecal calprotectin (Geertsema et al., 2026; Lorenzon Dos Santos et al., 2020; Tratenšek et al., 2024). Whereas CRP and fecal calprotectin primarily reflect inflammatory burden, redox biomarkers may also reflect oxidative tissue injury, antioxidant capacity, and restoration of redox homeostasis during treatment, potentially providing additional insight into biological responses to therapy (Geertsema et al., 2026; Lorenzon Dos Santos et al., 2020; Tratenšek et al., 2024). As IBD therapies target specific immune pathways, there is growing interest in identifying biomarkers that reflect both inflammatory control and restoration of redox homeostasis, thereby offering potential value in monitoring treatment response (Ding et al., 2016; Soendergaard et al., 2018).

Despite progress in therapeutic options for IBD management, a substantial proportion of patients fail to adequately respond to induction therapy (primary non-response), and many others lose response over time (secondary non-response), eventually requiring surgery (CD: 70%–90%, UC: 25%–30%) for stricturing, penetrating disease, or therapy-refractory inflammation (Kaplan, 2015). Early biochemical shifts in oxidative stress markers may accompany, or even precede, clinical or endoscopic improvement (Tratenšek et al., 2025). Following this hypothesis, integrating redox biomarkers with established clinical measures could improve stratification and help identify non-responders at an early therapeutic stage.

We have previously observed in an overlapping study with the same cohort that iron metabolism and redox biomarkers are intimately associated (Loveikyte et al., 2023). Hepcidin correlated strongly with iron indices but only weakly with markers of inflammation and oxidative stress, while oxidative stress appeared to be higher in patients with iron deficiency irrespective of inflammatory status. These findings suggest systemic redox alterations in IBD may, at least in part, reflect disturbances in iron handling rather than inflammation alone. Given that thiol-based antioxidants act as regulators of oxidative stress and iron is a key mediator of redox reactions, changes in iron and redox biomarkers could be expected during effective anti-inflammatory treatment (Bernstein et al., 2012; Alexdottir et al., 2022; Aksan et al., 2020).

In this exploratory prospective cohort study, we evaluated FTs, glutathione, cysteine, homocysteine, Trx1, inflammation and iron-related biomarkers in patients with IBD undergoing IFX and VEDO induction therapy. We hypothesized that active IBD is characterized by changes in thiol-based antioxidant capacity and Trx1, and that successful induction therapy is accompanied by distinct changes in these redox metabolites. Defining such a pattern could support more research into minimally invasive monitoring of inflammatory activity and facilitate earlier identification of treatment non-response.

## 2 Materials and methods

### 2.1 Study population and study design

In this prospective study, we included patients with an established diagnosis of CD, UC, or IBD unclassified (IBD-U) from the Dutch IBD Biobank, a prospectively maintained cohort containing detailed clinical data and stored biomaterials (Spekhorst et al, 2017). All adult biobank participants at the University Medical Center Groningen (UMCG) were screened between January and November 2021. Patients were eligible if they had clinically, biochemically, or endoscopically active IBD and initiated induction therapy with infliximab (IFX) or vedolizumab (VEDO). This study builds upon a previously conducted analysis within a partially overlapping cohort that focused on different circulating biomarkers in relation to treatment response (Loveikyte et al., 2023). In the present follow-up study, we specifically examined systemic redox markers. Serum samples were collected at predefined time points during induction therapy, namely at baseline (week 0), week 6, and week 14. We excluded patients treated with biologics for indications other than active IBD, as well as those with serum samples available only at week 6 of induction therapy. Additional exclusions were applied for comorbidities known to affect systemic inflammation or redox biology, including heart failure, liver cirrhosis, chronic obstructive pulmonary disease, hemoglobinopathies, autoimmune hemolytic anemia, myelodysplastic syndrome, end-stage renal disease (estimated glomerular filtration rate <30 mL/min/1.73 m^2^), and active malignancy other than nonmelanoma skin cancer. Pregnant or lactating women, patients with active infection, and those who had undergone major surgery (e.g., laparoscopic colectomy) within 6 weeks prior to induction therapy were also excluded.

### 2.2 Data collection

Demographic, anthropometric, and detailed clinical information were obtained from medical records and recorded at the start of induction therapy (Loveikyte et al., 2023). Biochemical parameters were collected at baseline, week 6, and week 14 of IFX or VEDO therapy. Baseline Montreal classification and information on clinical, biochemical, and endoscopic or radiologic disease activity were also retrieved from medical records (Table 1).

**TABLE 1:** Baseline demographic, clinical characteristics of the study population.

Variable	Total	CD	UC	p-value^a^
Disease subtype, n	58 (100.0%)	31 (53.4%)	27 (46.6%)	—
Biological type, n (%)				<0.05
Infliximab	37 (63.8%)	26 (83.9%)	11 (40.7%)	​
Vedolizumab	21 (36.2%)	5 (16.1%)	16 (59.3%)	
Demographics				
Age (years)	42.67 [31.79–55.00]	40.82 [24.72–52.33]	47.00 [37.00–55.81]	**<0.05**
Female, n	24 (41.4%)	17 (54.8%)	7 (25.9%)	**<0.05**
Anthropometrics				
BMI (kg/m^2^)	24.99 [22.05–29.70]	23.20 [21.06–29.12]	25.77 [24.61–29.70]	0.07
Smoking status, n^b^				0.48
Never	15 (28.3%)	7 (24.1%)	8 (33.3%)	​
Former	30 (56.6%)	16 (55.2%)	14 (58.3%)	
Current	8 (15.1%)	6 (20.7%)	2 (8.3%)	
Disease duration (years)	9.68 [5.04–19.15]	10.11 [6.34–21.10]	7.95 [4.18–18.37]	0.28
Montreal classification at baseline				
Age of onset, n				0.06
Age <17 (A1)	8. (13.8%)	7 (22.6%)	1 (3.7%)	​
Age 17–40 (A2)	34 (58.6%)	18 (58.1%)	16 (59.3%)	
Age >40 (A3)	16 (27.6%)	6 (19.4%)	10 (37.0%)	
Disease location, n				
Ileal (L1)	—	12 (38.7%)	—	—
Colonic (L2)	—	2 (6.5%)	—	—
Ileocolonic (L3)	—	17 (54.8%)	—	—
Upper GI-tract (L4)	—	—	—	—
Disease behavior, n				
Non-stricturing, non-penetrating (B1)	—	19 (61.3%)	—	—
Stricturing (B2)	—	7 (22.6%)	—	—
Penetrating (B3)	—	5 (16.1%)	—	—
Disease extent, n				
Proctitis (E1)	—	—	4 (15.4%)	—
Left-sided colitis (E2)	—	—	7 (26.9%)	—
Pancolitis (E3)	—	—	15 (57.7%)	—
Disease severity, n				
UC in remission (S0)	—	—	1 (4.0%)	—
Mildly active UC (S1)	—	—	6 (24.0%)	—
Moderately active UC (S2)	—	—	15 (60.0%)	—
Severely active UC (S3)	—	—	3 (12.0%)	—
Surgical history				
Colonic resection, n	2 (3.4%)	1 (3.2%)	1 (3.7%)	0.99
Ileocecal resection, n	9 (15.5%)	9 (29.0%)	—	—
Medical history				
Naive to biologicals, n	35 (60.3%)	17 (54.8%)	18 (66.7%)	0.36
Aminosalicylates, n	20 (34.5%)	3 (9.7%)	17 (63.0%)	**<0.05**
Immunomodulators, n				
None	18 (31.0%)	11 (35.5%)	7 (25.9%)	0.32
Thiopurines	38 (65.5%)	18 (58.1%)	20 (74.1%)	
Methotrexate	2 (3.4%)	2 (6.5%)	—	
Local or systemic steroids	32 (55.2%)	15 (48.4%)	17 (63.0%)	
Disease activity				
HBI^b^	4.5 [2–9]	4.5 [2–9]	—	—
SCCAI^b^	5 [3–7]	—	5 [3–7]	—
CRP, mg/L	2.85 [1.30–8.40]	2.90 [1.50–15.00]	2.70 [0.80–5.00]	0.63
fCal, mg/kg^b^	842.5 [288–2,190]	740 [235–2,200]	1,021 [400–2,130]	0.92
Radiological disease activity^b^	10 (17.2%)	10 (32.2%)	—	—
Endoscopic disease activity^b^	30 (51.7%)	13 (41.9%)	17 (63.0%)	0.11
Endoscopic Mayo score^b^				
Mayo 1	—	—	2 (7.4%)	—
Mayo 2	—	—	7 (25.9%)	—
Mayo 3	—	—	8 (29.6%)	—
Endoscopic SES-CD score				
Mild	—	7 (22.6%)	—	—
Moderate	—	2 (6.5%)	—	—
Severe	—	1 (3.2%)	—	—
Treatment outcomes				
Short-term response after 14 weeks, n (%)				
Responder	49 (84.5%)	25 (80.6%)	24 (88.9%)	0.48
Non-responder	9 (15.5%)	6 (19.4%)	3 (11.1%)	
Short-term response IFX after 14 weeks, n (%)				
Responder	34 (91.9%)	23 (88.5%)	11 (100.0%)	0.54
Non-responder	3 (8.1%)	3 (11.5%)	—	
Short-term response VEDO after 14 weeks, n (%)				
Responder	15 (71.4%)	2 (40.0%)	13 (81.3%)	0.11
Non-responder	6 (28.6%)	3 (60.0%)	3 (18.7%)	
Long-term response at 1 year, n (%)^b^				
Responder	40 (75.5%)	23 (76.7%)	17 (73.9%)	0.82
Non-responder	13 (24.5%)	7 (23.3%)	6 (26.1%)	
Long-term response IFX at 1 year, n (%)^b^				
Responder	31 (83.8%)	22 (84.6%)	9 (81.8%)	0.83
Non-responder	6 (16.2%)	4 (15.4%)	2 (18.2%)	
Long-term response VEDO at 1 year, n (%)^b^				
Responder	9 (56.3%)	1 (25.0%)	8 (66.7%)	0.26
Non-responder	7 (43.7%)	3 (75.0%)	4 (33.3%)	

^a^ Wilcoxon rank sum test; Pearson’s Chi-squared test; Fisher’s exact test. Data is presented as median [interquartile range] or n (%).

^b^ Missing data.

Abbreviations: BMI, body mass index; CD, Crohn’s disease; CRP, C-reactive protein; fCal, fecal calprotectin; GI, gastrointestinal; HBI, Harvey-Bradshaw Index; IFX, infliximab; SCCAI, simple clinical colitis activity index; SES-CD, Simple Endoscopic Score for Crohn’s Disease; UC, ulcerative colitis; VEDO, vedolizumab.

### 2.3 Study outcomes and definitions

Disease activity was evaluated at baseline and the end of the induction therapy (i.e., week 14). Clinical disease activity was evaluated using the Simple Clinical Colitis Activity Index for patients with UC or IBD-U and the Harvey-Bradshaw Index for patients with CD (Harvey et al., 1980; Walmsley et al, 1998). Biochemical disease activity was evaluated by either fCal or CRP measurements (defined as fCal >150 mg/kg and/or CRP >5 mg/L). Endoscopic disease activity was assessed using the Mayo endoscopic subscore for UC and IBD-U; the Simple Endoscopic Score for Crohn’s Disease (SES-CD) was used for CD. Due to the retrospective nature of the study, endoscopic data were retrieved from medical records at baseline (no more than 90 days before the start of therapy) and after induction therapy (but no more than 90 days after week 14 of therapy). Endoscopic scores were retrieved from the endoscopy reports or assessed by two clinical investigators based on available endoscopic images and endoscopy reports written by certified gastroenterologists employed at the UMCG. For patients who underwent capsule endoscopy, disease activity was not evaluated using Mayo or SES-CD scores but was noted as the presence or absence of endoscopic disease activity. Data on available radiologic disease activity were extracted from medical records; IBD disease activity was evaluated by certified radiologists employed at the UMCG who were not involved in the study.

Response to IFX or VEDO induction therapy was assessed at week 14 (including for FTs, despite the absence of week 14 FT measurements). Patients were classified as responders if they showed normalization or a ≥3-point reduction in the Harvey-Bradshaw Index (for CD) or the Simple Clinical Colitis Activity Index (for UC), or if the Physician’s Global Assessment (PGA) documented clinical, biochemical, radiologic, or endoscopic improvement. When the PGA was not explicitly reported, two clinical investigators independently evaluated treatment response using predefined criteria: improvement in clinical scores (normalization or ≥3-point decrease), biochemical improvement (normalization or ≥40% reduction in fCal and/or normalization of CRP), or endoscopic improvement (≥1-point decrease in endoscopic score). Patients who did not meet these criteria, or who discontinued induction therapy before week 14 for reasons other than adverse effects, were classified as short-term non-responders. Long-term response was defined as continuation of IFX or VEDO treatment beyond 1 year. Patients who continued biologic therapy beyond 1 year were classified as long-term responders, whereas patients who discontinued biologic therapy within 1 year were classified as long-term non-responders. This outcome reflects treatment durability rather than a direct objective measure of disease response.

### 2.4 Laboratory measurements

Serum samples taken at baseline, week 6, and week 14 of the induction therapy were stored at −80 °C until analysis. Serum FT levels were measured in triplicate with colorimetric detection using previously described methods (Ellman et al., 1959; Hu et al., 1994). Serum levels of glutathione (Catalogue No: abx257145), homocysteine (Catalogue No: abx576699), cysteine (Catalogue No: abx515392) and Trx1 (Catalogue No: EH451RB) were quantified by enzyme-linked immunosorbent assays (ELISAs; Abbexa Ltd., Cambridge, United Kingdom, and Thermo Fisher Scientific, Waltham, MA, United States. Appropriate sample dilutions were determined by pilot experiments described in a previous study (Geertsema et al, 2026).

### 2.5 Statistical analysis

Demographic and clinical characteristics of the study population were presented as means ± standard deviations (SD), proportions (*n*) with corresponding percentages (%) or medians with interquartile range (IQR). Normality assessment was performed by visual inspection of normal probability plots (Q-Q) and histograms. Paired analyses were performed using paired *t*-tests or Wilcoxon’s signed rank tests to evaluate differences between two time points. In addition, linear mixed-effects models were used to assess overall longitudinal changes in biomarker levels across the three sampling time points (week 0, week 6, and week 14), accounting for within-subject correlations. To compare biochemical parameters and changes in parameters between independent (sub)groups, we used Kruskal-Wallis tests, Mann-Whitney *U*-tests, chi-square, or independent-sample *t*-tests as appropriate. Spearman’s rank correlation coefficients (ρ) were used to determine correlations between variables.

Furthermore, univariable logistic regression analyses were performed to identify variables that were associated with the induction therapy and long-term biologic outcomes. For biomarker analyses, three logistic regression models were constructed: Model 1 was unadjusted; Model 2 was adjusted for sex, age, BMI, and smoking status; and Model 3 was additionally adjusted for biologic-naïve status and concomitant immunomodulator use. Biomarker levels were standardized using z-scores, with effect estimates expressed as odds ratios per 1 standard deviation increase. Given the limited sample size and small number of non-responders, the multivariable models may be susceptible to model instability and overfitting and should therefore be interpreted cautiously. Two-tailed p-values <0.05 were considered statistically significant; the Benjamini-Hochberg procedure was used to adjust for multiple testing, considering significance under a false discovery rate of 5% (0.05). Data analysis was performed using R (version 4.5.0, Vienna, Austria), and data visualization was performed using RStudio (version: 2025.05.1 + 513, JJ Allaire, Boston, MA, United States).

## 3 Results

### 3.1 Baseline characteristics of the study population

A total of 58 patients with IBD initiating biologic therapy were included, consisting of 31 patients with CD (53.4%) and 27 with UC (46.6%). Baseline demographic and clinical characteristics are summarized in Table 1. Patients with UC were significantly older than those with CD (median 47.0 vs. 40.8 years, p < 0.05) and were less often female (25.9% vs. 54.8%, p < 0.05). BMI tended to be higher in UC compared with CD, although this did not reach statistical significance (p = 0.07). Smoking status, disease duration, and prior surgical history were comparable between groups. Montreal classification patterns differed between disease types by design: patients with CD predominantly exhibited ileocolonic involvement (L3, 54.8%) and non-stricturing, non-penetrating behavior (B1, 61.3%), whereas UC patients most commonly presented with pancolitis (E3, 57.7%) and moderate endoscopic severity (S2, 60.0%).

IFX was more frequently prescribed in CD (26 patients with CD (83.9%) vs. 11 patients with UC (40.7%), p < 0.05), whereas VEDO was more often initiated in UC (5 patients with CD (16.1%) vs. 16 patients with UC (59.3%), p < 0.05). Short-term response rates did not differ between CD and UC (80.6% vs. 88.9%, p = 0.48), nor when analyzed separately for IFX- and VEDO-treated patients (respectively, p = 0.54 and p = 0.11). Similarly, long-term response rates at 1 year were comparable between patients with CD and UC.

### 3.2 Inflammatory and redox biomarker profiles during induction therapy

Changes in inflammation- and redox biomarkers during induction therapy with IFX or VEDO are shown in Table 2 (Supplementary Tables S1, S2). In the overall IBD cohort, median cysteine levels decreased from 3,247 μg/mL (IQR 2,735–4,045) at baseline to 3,093 μg/mL (IQR 2,575–3,787) at week 6 (adjusted p < 0.05) but returned to baseline levels at week 14 (3,389 μg/mL; IQR 2,608–4,019; adjusted p = 0.76). No significant changes were observed for homocysteine, FTs, glutathione and Trx1 during induction therapy.

**TABLE 2:** Changes in inflammation- and redox biomarkers during induction therapy with either IFX or VEDO in patients with IBD.

Biomarker	Baseline (n = 58)	Week 6 (n = 58)	Week 14 (n = 58)	Δ baseline to week 6	Δ baseline to week 14
Cysteine (µg/mL)	3,247 (2,735–4,045)	3,093 (2,575–3,787)	3,389 (2,608–4,019)	<0.05	0.76
Homocysteine (ng/mL)	3,476 (2,961–6,018)	3,428 (2,997–5,643)	3,568 (3,061–6,418)	0.79	0.35
Free thiols (µmol/L)	233.90 (206.65–270.36)	229.71 (213.83–286.50)	—	0.40	—
Glutathione (µg/mL)	81.76 (60.92–98.49)	75.27 (59.78–97.47)	78.71 (65.44–99.44)	0.23	0.82
Trx1 (ng/mL)	119.30 (51.02–271.87)	132.70 (48.90–493.30)	126.82 (51.44–222.59)	0.41	0.68
CRP (mg/L)	2.85 (1.35–7.80)	1.40 (0.50–3.95)	1.25 (0.50–4.00)	**<0.05**	**<0.05**
Fecal calprotectin (mg/kg)	842.50 (289.00–2,185)	—	120.00 (41.00–300.00)	—	**<0.05**

IFX					
Biomarker	Baseline (n = 37)	Week 6 (n = 37)	Week 14 (n = 37)	Δ baseline to week 6	Δ baseline to week 14
Cysteine (µg/mL)	3,284 (2,805–4,002)	2,781 (2,353–3,697)	3,364 (2,569–3,870)	<0.05	0.75
Homocysteine (ng/mL)	3,427 (2,966–4,985)	3,260 (2,956–5,038)	3,518 (3,042–5,996)	0.19	0.75
Free thiols (µmol/L)	233.12 (206.41–266.34)	228.97 (211.41–286.75)	—	0.68	—
Glutathione (µg/mL)	82.90 (66.26–101.22)	75.30 (64.94–100.09)	78.87 (65.55–103.90)	0.19	0.88
Trx1 (ng/mL)	129.54 (53.57–317.50)	124.74 (73.54–524.67)	116.22 (47.81–225.79)	0.68	0.68
CRP (mg/L)	3.00 (1.50–8.40)	1.30 (0.40–2.20)	0.80 (0.30–2.30)	**<0.05**	**<0.05**
Fecal calprotectin (mg/kg)	2,020 (682.50–2,230)	—	120.00 (66.50–247.50)	—	0.38

VEDO					
Biomarker	Baseline (n = 21)	Week 6 (n = 21)	Week 14 (n = 21)	Δ baseline to week 6	Δ baseline to week 14
Cysteine (µg/mL)	3,135 (2,712–4,258)	3,330 (2,830–4,338)	3,393 (2,687–4,067)	0.86	0.99
Homocysteine (ng/mL)	3,575 (2,764–6,200)	3,934 (3,069–5,867)	3,725 (3,226–6,474)	0.38	0.18
Free thiols (µmol/L)	238.33 (207.37–275.56)	232.82 (217.09–280.41)	—	0.38	—
Glutathione (µg/mL)	68.86 (55.28–96.39)	75.25 (55.51–84.22)	76.80 (65.40–88.26)	0.67	0.99
Trx1 (ng/mL)	101.12 (34.94–195.33)	140.67 (29.38–399.17)	137.41 (59.26–189.70)	0.47	0.89
CRP (mg/L)	2.60 (1.30–6.00)	1.60 (1.20–7.00)	1.70 (0.80–8.00)	0.38	0.21
Fecal calprotectin (mg/kg)	420.00 (286.00–1,268.00)	—	121.50 (42.75–500.50)	—	0.21

Data is presented as median [interquartile range]. Two-tailed P values < 0.05 were considered statistically significant; the Benjamini-Hochberg procedure was used to adjust for multiple testing, adopting a 5% false discovery rate. Abbreviations: CRP, C-reactive protein; IBD, inflammatory bowel disease; IFX, infliximab; Trx1, Thioredoxin-1; VEDO, vedolizumab.

In contrast, inflammatory biomarkers declined during treatment. Median CRP levels decreased from 2.85 mg/L (IQR 1.35–7.80) at baseline to 1.40 mg/L (IQR 0.50–3.95) at week 6 and further to 1.25 mg/L (IQR 0.50–4.00) at week 14 (both adjusted p < 0.05). fCal levels were also significantly reduced from 843 mg/kg (IQR 289–2,185) at baseline to 120 mg/kg (IQR 41–300) at week 14 (adjusted p < 0.05).

When stratified by induction therapy, IFX-treated patients showed a significant early decrease in cysteine levels from 3,284 μg/mL (IQR 2,805–4,002) at baseline to 2,781 μg/mL (IQR 2,353–3,697) at week 6 (adjusted p < 0.05), with levels comparable to baseline at week 14 (3,364 μg/mL; IQR 2,569–3,870; adjusted p = 0.75). Other redox biomarkers, including homocysteine, FTs, glutathione, and Trx1, did not change significantly during IFX induction. CRP decreased significantly from 3.00 mg/L (IQR 1.50–8.40) at baseline to 1.30 mg/L (IQR 0.40–2.20) at week 6 and 0.80 mg/L (IQR 0.30–2.30) at week 14 (both adjusted p < 0.05). fCal declined from 2,020 mg/kg (IQR 682.50–2,230) at baseline to 120 mg/kg (IQR 66.50–247.50) at week 14, although this reduction did not reach statistical significance after multiple testing correction. Among VEDO-treated patients, no significant changes were observed in any of the assessed redox biomarkers. Cysteine levels were 3,135 μg/mL (IQR 2,712–4,258) at baseline, 3,330 μg/mL (IQR 2,830–4,338) at week 6, and 3,393 μg/mL (IQR 2,687–4,067) at week 14 (all adjusted p > 0.05). Homocysteine, FTs, glutathione, and Trx1 levels similarly remained stable. CRP decreased numerically from 2.60 mg/L (IQR 1.30–6.00) at baseline to 1.70 mg/L (IQR 0.80–8.00) at week 14, and fCal from 420 mg/kg (IQR 286–1,268) to 122 mg/kg (IQR 42.75–500.50), but neither change reached statistical significance.

### 3.3 Free thiol changes differ by response, while IFX non-responders exhibit persistently adverse redox markers

When comparing non-adjusted biomarker levels, none of the redox biomarkers differed significantly between baseline and week 14 within short-term responders or short-term non-responders, either overall or when stratified by IFX and VEDO (Table 3). In contrast, fCal decreased significantly among IFX-treated short-term responders from 2,130 mg/kg (IQR 805–2,260) at baseline to 120 mg/kg (IQR 66.50–247.50) at week 14 (p < 0.05).

**TABLE 3:** Changes in inflammation- and redox biomarkers in patients with IBD which were categorized as short-term responders or non-responders during induction therapy with either IFX or VEDO.

​	Short-term responders			Short-term non-responders			​
Biomarker	Baseline	Week 14	Paired analysis	Baseline	Week 14	Paired analysis	Δ between groups
Cysteine (µg/mL)	3,320 (2,712–4,051)	3,364 (2,597–4,068)	0.85	3,135 (2,905–3,226)	3,697 (2,733–3,852)	0.68	0.48
Homocysteine (ng/mL)	3,459 (2,976–6,227)	3,518 (3,042–6,335)	0.85	3,504 (2,635–4,997)	3,725 (3,226–6,474)	0.73	0.94
FTs (µmol/L)^a^	234.23 (208.87–271.41)	236.58 (217.28–287.93)	0.20	230.99 (204.71–244.96)	217.09 (197.63–223.73)	0.63	0.19
Glutathione (µg/mL)	81.10 (58.04–98.47)	76.80 (61.99–95.70)	0.85	91.08 (76.53–102.75)	88.61 (81.29–108.02)	0.82	0.94
Trx1 (ng/mL)	129.54 (50.88–271.87)	137.41 (47.81–212.99)	0.90	90.56 (78.14–412.42)	89.66 (77.38–344.83)	0.63	0.19
CRP (mg/L)	2.60 (1.20–5.00)	0.80 (0.50–2.30)	0.08	15.00 (2.80–27.00)	10.00 (1.80–51.00)	0.63	0.19
Fecal calprotectin (mg/kg)	1,110 (286.00–2,200)	106.50 (40.75–200.50)	**<0.05**	709.00 (512.50–810.00)	595.00 (105.00–2,310)	0.73	0.11

​	Short-term IFX responders			Short-term IFX non-responders			​
Biomarker	Baseline	Week 14	Paired analysis	Baseline	Week 14	Paired analysis	Δ between groups
Cysteine (µg/mL)	3,599 (2,944–4,023)	3,375 (2,576–4,095)	0.97	2,341 (1,891–2,783)	2,733 (2,492–3,207)	0.73	0.76
Homocysteine (ng/mL)	3,412 (2,967–4,353)	3,497 (2,948–4,268)	0.97	6,018 (4,423–9,836)	8,601 (5,824–8,979)	0.73	0.76
FTs (µmol/L)^a^	231.74 (207.02–264.77)	229.53 (211.79–286.50)	0.87	240.69 (175.35–279.09)	218.01 (160.13–260.33)	0.73	0.76
Glutathione (µg/mL)	82.63 (65.88–100.54)	78.79 (66.02–103.83)	0.97	91.08 (83.80–96.91)	90.68 (66.17–99.35)	0.73	0.76
Trx1 (ng/mL)	169.76 (55.07–362.10)	170.05 (48.40–248.02)	0.97	90.56 (68.29–104.61)	77.38 (38.94–79.26)	0.94	0.91
CRP (mg/L)	3.20 (1.27–7.55)	0.80 (0.30–2.30)	0.09	2.80 (2.20–14.90)	1.80 (1.15–42.40)	0.07	**<0.05**
Fecal calprotectin (mg/kg)	2,130 (805.00–2,260)	120.00 (66.50–247.50)	**<0.05**	682.50 (653.75–711.25)	—	—	—

​	Short-term VEDO responders			Short-term VEDO non-responders			​
Biomarker	Baseline	Week 14	Paired analysis	Baseline	Week 14	Paired analysis	Δ between groups
Cysteine (µg/mL)	2,984 (2,533–4,580)	3,268 (2,672–3,847)	0.57	3,143 (3,062–3,830)	3,832 (3,726–4,134)	0.63	0.35
Homocysteine (ng/mL)	3,725 (3,082–7,589)	4,054 (3,229–8,601)	0.46	3,070 (2,493–4,393)	3,512 (3,244–4,142)	0.44	0.74
FTs (µmol/L)^a^	238.55 (213.00–276.88)	258.87 (232.55–290.13)	0.26	219.18 (205.37–241.47)	215.46 (201.68–222.07)	0.63	0.27
Glutathione (µg/mL)	61.89 (52.03–88.52)	69.72 (57.02–81.47)	0.70	90.53 (72.29–108.85)	87.86 (82.74–121.33)	0.93	0.84
Trx1 (ng/mL)	101.12 (27.30–183.81)	110.66 (47.97–165.02)	0.95	248.83 (79.92–522.34)	258.34 (110.21–500.11)	0.44	0.27
CRP (mg/L)	2.20 (1.05–2.85)	1.40 (0.75–2.30)	0.57	20.00 (7.50–51.25)	20.00 (9.25–45.75)	0.80	0.92
Fecal calprotectin (mg/kg)	410.00 (247.00–1,364)	69.00 (41.00–170.00)	0.18	709.00 (400.00–880.00)	595.00 (105.00–2,310)	0.80	0.27

Data is presented as median [interquartile range]. Two-tailed P values < 0.05 were considered statistically significant. Within-group changes were analyzed using linear mixed-effects models with a random intercept for each patient. For each biomarker, estimated marginal means were used to obtain the time effect separately for responders and non-responders (paired comparison). Between-group differences in change were evaluated using the time × response interaction term. P-values were adjusted for multiple testing using the Benjamini-Hochberg procedure (FDR, 5%). Abbreviations: CRP, C-reactive protein; IBD, inflammatory bowel disease; IFX, infliximab; Trx1, Thioredoxin-1; VEDO, vedolizumab.

^a^ Week 6 for FTs, as no Week 14 FTs, measurements were taken.

For cysteine, mixed-effects analyses showed that IFX-treated short-term non-responders displayed lower estimated cysteine levels than IFX-treated short-term responders already at baseline (2,336 vs. 3,611 μg/mL), with this difference persisting at follow-up (week 14: 2,888 vs. 3,599 μg/mL), whereas VEDO-treated short-term responders and non-responders had comparable estimated baseline levels (Figures 1A, 2A). For homocysteine, IFX-treated short-term non-responders had higher estimated homocysteine levels than IFX-treated short-term responders at baseline (7,500 vs. 4,260 ng/mL; p < 0.05) and remained elevated over time (week 14: 7,002 vs. 4,278 ng/mL), whereas homocysteine levels in VEDO-treated patients were lower and more comparable between response groups (Figures 1B, 2B). For FTs, the overall change from baseline to week 6 was modest and not significant (estimated difference 7.17 μmol/L; p = 0.26). The interaction model showed a short-term response-dependent change, consistent with higher estimated FT levels at week 6 in short-term responders (IFX: 239–247 μmol/L; VEDO: 236–256 μmol/L) and lower levels in short-term non-responders (IFX: 223 to 208 μmol/L; VEDO: 226 to 207 μmol/L) (Figures 1C, 2C).

**FIGURE 1:**
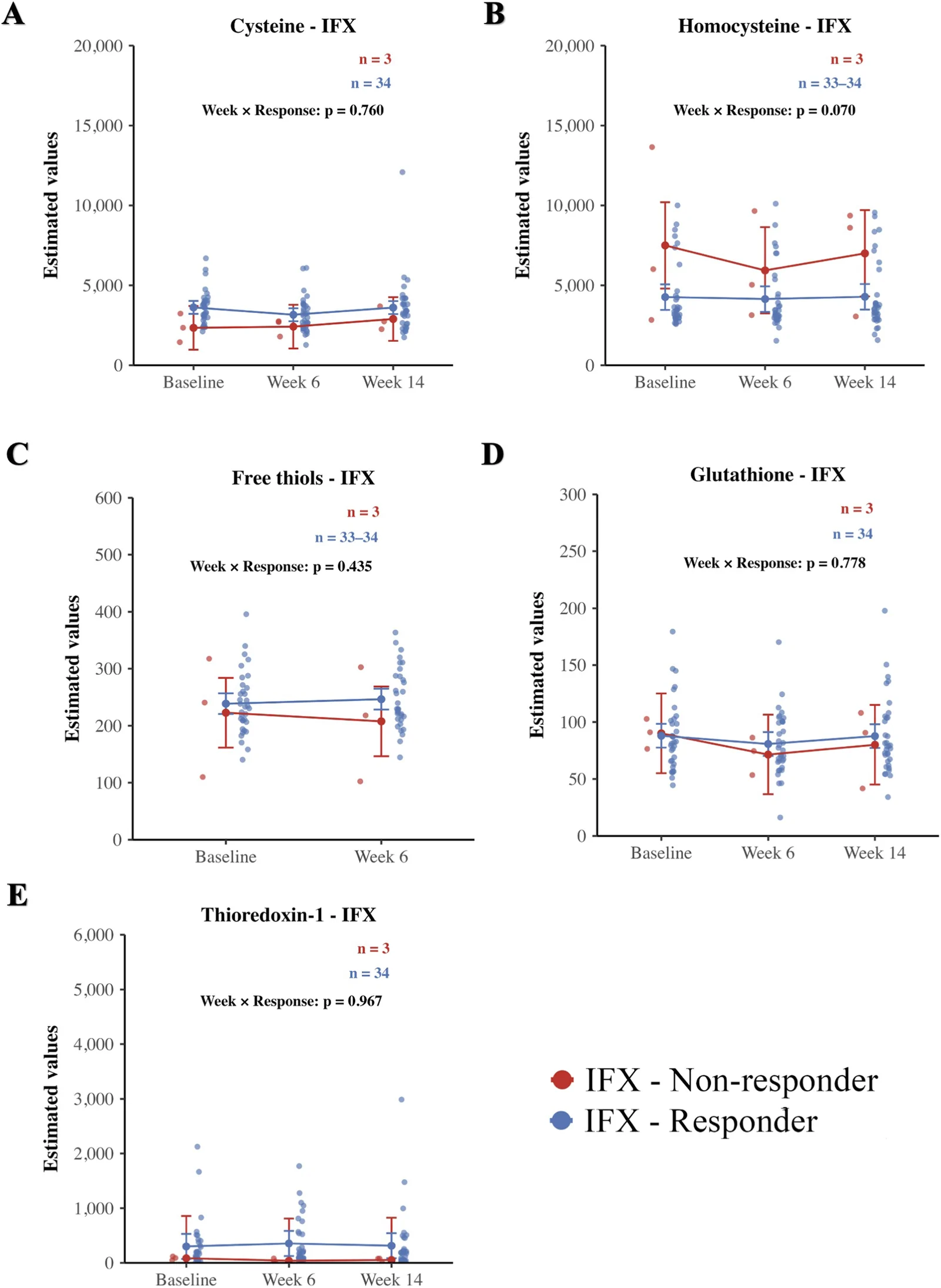
Estimated redox-related biomarker levels over time in IFX-treated patients stratified by clinical response at week 14 shows homocysteine with best performance. Estimated marginal means of biomarkers from linear mixed-effects models with a random intercept for each patient and fixed effects for time (baseline, week 6, and week 14), clinical short-term response (responder vs. non-responder), and their interaction for patients with IBD treated with IFX. **(A)** Estimated cysteine levels over time in patients with IBD treated with IFX. **(B)** Estimated homocysteine levels over time in patients with IBD treated with IFX. **(C)** Estimated free thiol (FT) levels over time in patients with IBD treated with IFX. **(D)** Estimated glutathione levels over time in patients with IBD treated with IFX. **(E)** Estimated thioredoxin-1 (Trx1) levels over time in patients with IBD treated with IFX. Time courses are shown separately for IFX short-term responders and IFX short-term non-responders. Individual patient data points, sample sizes, clearly defined error bars (95% confidence intervals), and time-by-response interaction p-values are shown. Abbreviations: 95% CI, 95% confidence interval; FTs, free thiols; Trx1, thioredoxin-1; IFX, infliximab; IBD, inflammatory bowel disease.

**FIGURE 2:**
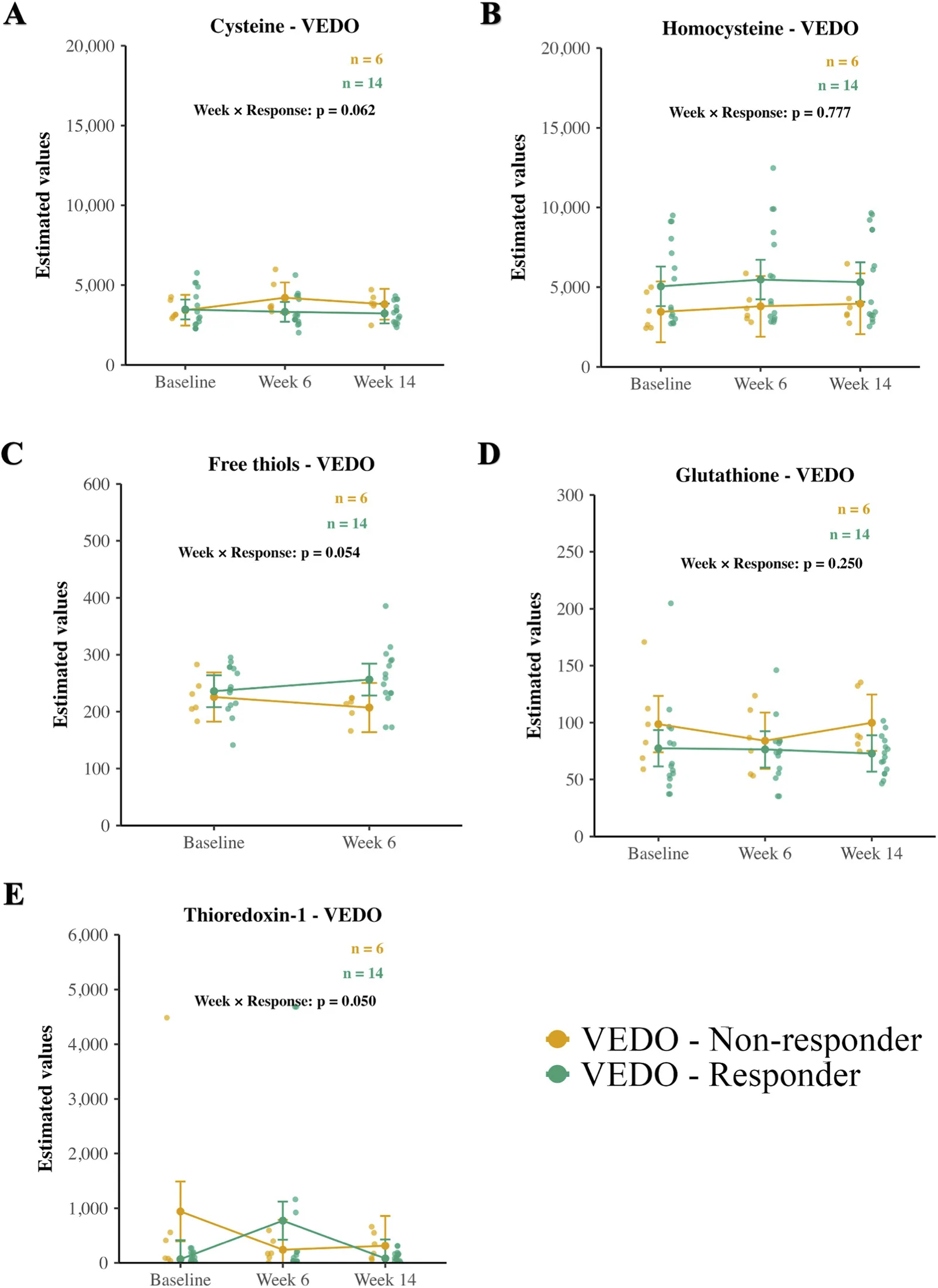
Estimated redox-related biomarker levels over time in VEDO-treated patients stratified by clinical response at week 14 shows clear response-associated changes for FTs and glutathione. Estimated marginal means of biomarkers from linear mixed-effects models with a random intercept for each patient and fixed effects for time (baseline, week 6, and week 14), clinical short-term response (responder vs. non-responder), and their interaction for patients with IBD treated with VEDO. **(A)** Estimated cysteine levels over time in patients with IBD treated with VEDO. **(B)** Estimated homocysteine levels over time in patients with IBD treated with VEDO. **(C)** Estimated free thiol (FT) levels over time in patients with IBD treated with VEDO. **(D)** Estimated glutathione levels over time in patients with IBD treated with VEDO. **(E)** Estimated thioredoxin-1 (Trx1) levels over time in patients with IBD treated with VEDO. Time courses are shown separately for VEDO short-term responders and VEDO short-term non-responders. Individual patient data points, sample sizes, clearly defined error bars (95% confidence intervals), and time-by-response interaction p-values are shown. Abbreviations: 95% CI, 95% confidence interval; FTs, free thiols; Trx1, thioredoxin-1; VEDO, vedolizumab; IBD, inflammatory bowel disease.

When examining the odds of short-term response at week 14, baseline cysteine demonstrated the strongest positive association, with higher levels associated with increased odds of response across all models; however, this association was not statistically significant after multivariable adjustment (Model 5: OR 3.25, 95% CI 0.49–29.36, p = 0.29; Table 4). Baseline homocysteine, FTs, glutathione, and Trx1 showed no consistent associations with short-term response (Figures 1B-E, 2B-E). Analyses stratified by induction therapy (IFX and VEDO) did not reveal materially different associations compared with the overall cohort (Supplementary Tables S3, S4).

**TABLE 4:** Plasma biomarker levels and the chance for short-term and long-term response to induction therapy with IFX/VEDO.

​	Short-term response	Long-term response
Model	OR per SD of biomarker	OR per SD of biomarker
Cysteine (baseline)		
Model 1	1.84 [0.75–4.54], p = 0.18	0.93 [0.50–1.73], p = 0.81
Model 2	3.84 [0.72–20.56], p = 0.12	1.53 [0.66–3.54], p = 0.32
Model 3	3.25 [0.49–21.71], p = 0.22	1.35 [0.45–4.08], p = 0.59
Model 4	3.87 [0.49–30.26], p = 0.20	1.38 [0.45–4.27], p = 0.57
Model 5	3.25 [0.36–29.36], p = 0.29	1.30 [0.39–4.29], p = 0.67
Homocysteine (baseline)		
Model 1	0.93 [0.47–1.87], p = 0.85	0.96 [0.51–1.79], p = 0.90
Model 2	1.08 [0.47–2.44], p = 0.86	0.75 [0.33–1.70], p = 0.49
Model 3	1.10 [0.41–2.94], p = 0.85	1.07 [0.36–3.16], p = 0.91
Model 4	1.04 [0.35–3.05], p = 0.95	1.07 [0.36–3.17], p = 0.90
Model 5	1.25 [0.21–7.26], p = 0.80	1.19 [0.38–3.69], p = 0.76
Free thiols (baseline)		
Model 1	1.34 [0.63–2.83], p = 0.44	1.06 [0.56–2.00], p = 0.86
Model 2	0.90 [0.39–2.11], p = 0.81	0.71 [0.33–1.53], p = 0.38
Model 3	0.77 [0.25–2.33], p = 0.64	0.70 [0.28–1.76], p = 0.45
Model 4	0.86 [0.25–2.92], p = 0.80	0.69 [0.27–1.78], p = 0.44
Model 5	—^a^	0.53 [0.17–1.66], p = 0.27
Glutathione (baseline)		
Model 1	0.74 [0.39–1.41], p = 0.35	0.91 [0.49–1.69], p = 0.77
Model 2	0.57 [0.22–1.47], p = 0.24	1.16 [0.53–2.53], p = 0.72
Model 3	0.69 [0.19–2.51], p = 0.58	2.18 [0.58–8.21], p = 0.25
Model 4	0.58 [0.12–2.72], p = 0.49	2.93 [0.61–14.09], p = 0.18
Model 5	—^a^	3.19 [0.53–19.31], p = 0.21
Thioredoxin-1 (baseline)		
Model 1	0.65 [0.36–1.18], p = 0.16	0.60 [0.30–1.22], p = 0.16
Model 2	0.60 [0.27–1.34], p = 0.21	0.71 [0.26–1.96], p = 0.51
Model 3	0.86 [0.29–2.53], p = 0.78	0.46 [0.05–4.11], p = 0.49
Model 4	0.98 [0.32–3.04]. p = 0.98	0.47 [0.05–4.22], p = 0.50
Model 5	—^a^	0.40 [0.04–4.06], p = 0.44

Odds ratios with 95%-confidence intervals obtained from binary logistic regression models. Odds ratios are expressed per 1 standard deviation. Univariable models are without any correction for confounders. Model 1 is crude (unadjusted); Model 2 is Model 1 additionally adjusted for age, sex, BMI, and smoking status; Model 3 is Model 2 with further adjustment for biologic naïve and immunomodulators usage. Model 4 is Model 3 with further adjustment for disease type. Model 5 is Model 4 with baseline CRP. P-values <0.05 were considered statistically significant. Abbreviations: OR, odds ratio; SD, standard deviation; 95%CI, 95% confidence interval.

^a^ Estimates could not be reliably calculated because of model instability.

### 3.4 Baseline FTs and glutathione changes relate to long-term response

When examining biomarkers levels in relation to long-term response, cysteine, homocysteine, and thioredoxin-1 levels showed only modest changes over time, with largely overlapping trajectories between long-term responders and long-term non-responders, irrespective of induction therapy (Figures 3, 4).

**FIGURE 3:**
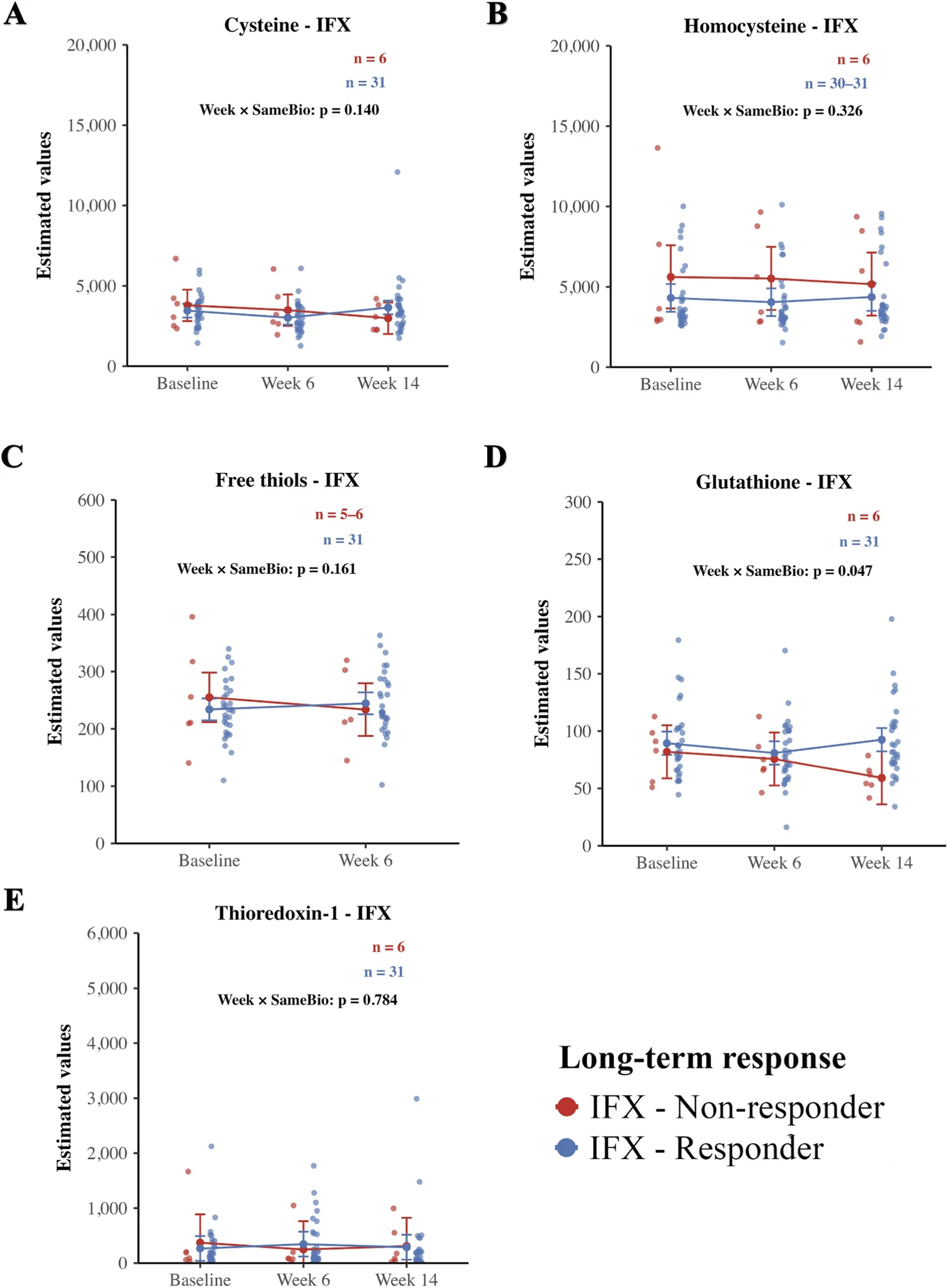
Estimated redox-related biomarker levels over time in IFX-treated patients stratified by biologic sustained treatment for at least 1 year. Estimated marginal means of biomarkers from linear mixed-effects models with a random intercept for each patient and fixed effects for time (baseline, week 6, and week 14), long-term response (responder vs. non-responder), and their interaction for patients with IBD treated with IFX. **(A)** Estimated cysteine levels over time in patients with IBD treated with IFX. **(B)** Estimated homocysteine levels over time in patients with IBD treated with IFX. **(C)** Estimated free thiol (FT) levels over time in patients with IBD treated with IFX. **(D)** Estimated glutathione levels over time in patients with IBD treated with IFX. **(E)** Estimated thioredoxin-1 (Trx1) levels over time in patients with IBD treated with IFX. Time courses are shown separately for IFX long-term responders and IFX long-term non-responders. Individual patient data points, sample sizes, clearly defined error bars (95% confidence intervals), and time-by-response interaction p-values are shown. Abbreviations: 95% CI, 95% confidence interval; FTs, free thiols; Trx1, thioredoxin-1; IFX, infliximab; IBD, inflammatory bowel disease.

**FIGURE 4:**
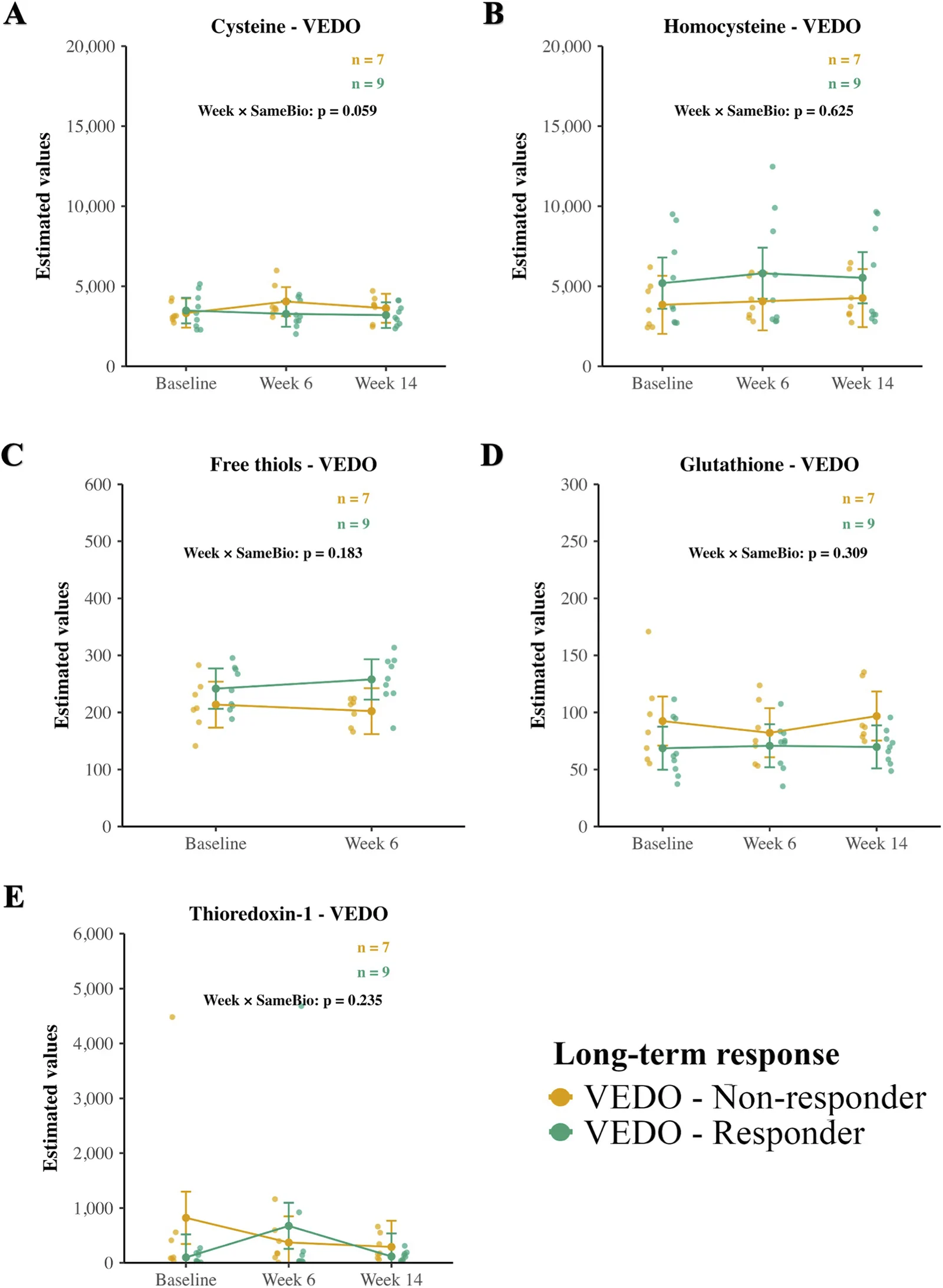
Estimated redox-related biomarker levels over time in VEDO-treated patients stratified by biologics sustained treatment for at least 1 year. Estimated marginal means of biomarkers from linear mixed-effects models with a random intercept for each patient and fixed effects for time (baseline, week 6, and week 14), long-term response (responder vs. non-responder), and their interaction for patients with IBD treated with VEDO. **(A)** Estimated cysteine levels over time in patients with IBD treated with VEDO. **(B)** Estimated homocysteine levels over time in patients with IBD treated with VEDO. **(C)** Estimated free thiol (FT) levels over time in patients with IBD treated with VEDO. **(D)** Estimated glutathione levels over time in patients with IBD treated with VEDO. **(E)** Estimated thioredoxin-1 (Trx1) levels over time in patients with IBD treated with VEDO. Time courses are shown separately for VEDO long-term responders and VEDO long-term non-responders. Individual patient data points, sample sizes, clearly defined error bars (95% confidence intervals), and time-by-response interaction p-values are shown. Abbreviations: 95% CI, 95% confidence interval; FTs, free thiols; Trx1, thioredoxin-1; VEDO, vedolizumab; IBD, inflammatory bowel disease.

In contrast, FT levels demonstrated a consistent divergence according to long-term response across both induction therapies (Figures 3C, 4C). While overall changes from baseline to week 6 were modest (baseline: 236 μmol/L; week 6: 243 μmol/L), long-term responders showed increasing estimated FT levels between baseline and week 6, whereas long-term non-responders exhibited decreasing FT levels over the same period. These changes were observed in both IFX- and VEDO-treated patients and was supported by a trend toward a week-by-long-term response interaction (p = 0.064). Trx1 levels showed no clear temporal or group-specific differences over time (Figure 3E).

For glutathione, overall temporal changes were modest at the cohort level (baseline: 86.7 μg/mL; week 6: 79.7 μg/mL; week 14: 84.9 μg/mL; p for time = 0.14) (Figures 3D, 4D). However, among IFX-treated long-term non-responders, estimated glutathione levels declined over time, with a decrease of approximately 22.8 μg/mL from baseline to week 14. In contrast, IFX-treated long-term responders showed an increase of approximately 25.8 μg/mL over the same period, resulting in a clear separation of levels by week 14. Consistent with this longitudinal change, baseline glutathione showed a not statistically significant association with long-term response in multivariable logistic regression analyses (Model 5: OR 3.19 per SD increase, 95% CI 0.53–19.31; Table 4).

### 3.5 Glutathione and FTs show (inverse) associations with iron and TSAT after induction therapy

We examined the relation between iron metabolism and redox biomarkers (Figure 5A). At baseline, FTs showed no significant associations with iron or transferrin saturation (TSAT) (iron: Figure 5B; TSAT; Figure 5D, all p > 0.05). After induction therapy (week 6), only weak and non-significant associations were observed between FTs and iron (r = 0.25, p = 0.06) (Figure 5C) and TSAT (r = 0.19, p = 0.16) (Figure 5E). Glutathione showed no significant association with iron (r = −0.18, p = 0.17) (Figure 5F) or TSAT (r = −0.20, p = 0.14) (Figure 5H) at baseline. In contrast to FT, glutathione became significantly and moderately inversely correlated with both iron (r = −0.46, p = 0.0006) (Figure 5G) and TSAT (r = −0.50, p = 0.0004) (Figure 5I) following induction therapy (week 14), suggesting a therapy-associated shift in the relationship between systemic iron availability and intracellular redox status.

**FIGURE 5:**
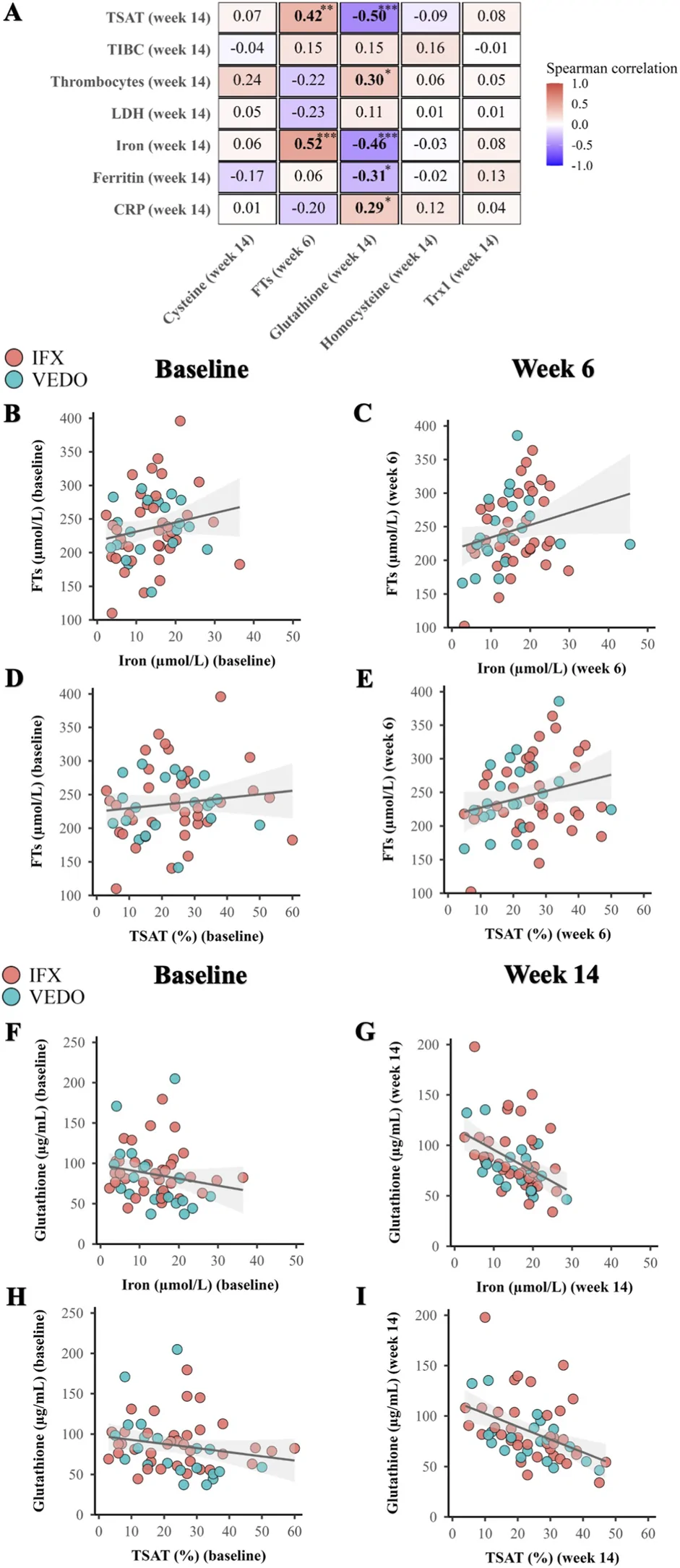
Associations between redox-related biomarkers and systemic iron parameters during biologics induction therapy in IBD. **(A)** Heatmap showing Spearman correlation coefficients between redox-related biomarkers (glutathione at week 14, Trx1 at week 14, cysteine at week 14, homocysteine at week 14, and free thiols (FTs) at week 6) and systemic iron parameters (thrombocytes, TSAT, TIBC, ferritin, iron, CRP, and LDH at week 14). **(B)** Scatterplot of FTs (baseline) versus iron (baseline). **(C)** Scatterplot of FTs (week 6) versus iron (week 6). **(D)** Scatterplot of FTs (baseline) versus TSAT (baseline). **(E)** Scatterplot of FTs (week 6) versus TSAT (week 6). **(F)** Scatterplot of glutathione (baseline) versus iron (baseline). **(G)** Scatterplot of glutathione (week 14) versus iron (week 14). **(H)** Scatterplot of glutathione (baseline) versus TSAT (baseline). **(I)** Scatterplot of glutathione (week 14) versus TSAT (week 14). In **(B–I)**, points are colored by induction therapy (IFX or VEDO), and shaded areas indicate 95% confidence intervals from linear regression fits. Asterisks denote statistical significance: *p < 0.05, **p < 0.005, ***p < 0.0005. Abbreviations: CRP, C-reactive protein; FTs, free thiols; GSH, glutathione; IBD, inflammatory bowel disease; IFX, infliximab; LDH, lactate dehydrogenase; TIBC, total iron-binding capacity; TSAT, transferrin saturation; VEDO, vedolizumab.

## 4 Discussion

In this study, we demonstrate that systemic redox biomarkers show some suggested associations in relation to short-term response, long-term response, and iron metabolism during induction therapy with IFX or VEDO in patients with IBD. At the cohort level, redox biomarkers remained largely unchanged during induction therapy, while inflammatory markers decreased significantly, particularly with IFX treatment, underscoring a partial dissociation between inflammatory control and systemic redox status. When stratified by short-term and long-term response, selected redox markers showed numerical differences between response groups, including lower baseline cysteine and persistently higher homocysteine levels in IFX-treated short-term non-responders, as well as divergent FT and glutathione trajectories according to long-term response. In addition, mainly glutathione and FTs showed strong inverse associations with iron and transferrin saturation after induction therapy, indicating a dynamic interaction between intracellular redox balance and systemic iron availability that became apparent only following effective treatment. Collectively, these findings suggest that systemic redox biomarkers may reflect biologically relevant processes related to short-term and long-term response and iron handling, with more heterogeneous effects observed in IFX-treated rather than VEDO-treated patients.

Redox- and iron-related biomarkers exhibited subtle, heterogeneous, and response-dependent changes compared with the reductions observed in inflammatory markers, particularly among IFX-treated patients. Lower cysteine levels in IFX-treated short-term non-responders were evident at baseline and throughout induction therapy, while persistently elevated homocysteine levels further characterized this subgroup, consistent with a less favorable systemic redox profile. Although causal mechanisms cannot be inferred from the present data, these changes may reflect alterations in sulfur amino acid metabolism or an increased oxidative burden leading to reduced cysteine availability and homocysteine accumulation under conditions of ongoing oxidative stress (Chiang et al., 2022; Perna et al., 2003; Mahmoudi et al 2025). Thus, from a clinical perspective, lower cysteine and higher homocysteine levels may indicate a higher systemic oxidative burden that is less amenable to IFX induction therapy. Measurement of these redox biomarkers could therefore support early identification of patients at risk for non-response or suboptimal sustained biologics continuation.

FTs displayed the most consistent and biochemically interpretable numerical changes over time, despite being assessed only at baseline and week 6. As circulating markers of the extracellular thiol redox pool, higher FT levels generally reflect a more favorable redox balance, whereas lower levels indicate increased oxidative stress. In line with this interpretation, FT levels increased during induction therapy in short-term responders treated with either IFX or VEDO, while they tended to decline in short-term non-responders. Importantly, this divergence was not limited to short-term response: FT levels increased during induction therapy in long-term responders but decreased in long-term non-responders, irrespective of induction therapy. This may suggest in our opinion that early induction-phase changes in circulating FTs may reflect systemic redox resilience or recovery, which in turn appears to be associated with sustained therapeutic benefit (Bourgonje et al., 2019; Bohra et al., 2025).

Not all investigated redox biomarkers showed consistent changes during induction therapy or differences between responders and non-responders. This is not unexpected, as the selected biomarkers represent different components of the redox system, including extracellular antioxidant capacity, intracellular thiol metabolism, and redox signaling (Bourgonje et al., 2020; Cortese-Krott et al., 2017). These biomarkers are not expected to respond uniformly to changes in inflammatory activity, and some may primarily reflect complementary biological processes, such as iron metabolism or antioxidant defense, rather than inflammation alone (Daniel et al 2020; Mahmoudi et al., 2025). In our cohort, glutathione correlated negatively with both TSAT and iron levels after induction therapy, but not at baseline, extending previous observations linking higher circulating glutathione levels to disease activity and oxidative stress in IBD (Geertsema et al., 2026). Our data suggest a potential association between glutathione and iron metabolism, as this pattern was not observed for other redox biomarkers. The absence of this association at baseline and its emergence after induction therapy may indicate that the relationship becomes more apparent once inflammatory activity is reduced. However, residual confounding by inflammation cannot be excluded. In contrast, FTs showed associations with iron parameters in the opposite direction, which is biologically plausible given their role as extracellular redox buffers and their strong dependence on albumin-bound thiol availability (Bourgonje et al., 2019; Bohra et al., 2025). One possible explanation for this inverse association is the close functional coupling between glutathione and iron metabolism (Daniel et al., 2020; Shi et al., 2024). Glutathione is required for intracellular iron trafficking and iron-sulfur cluster biogenesis, and previous studies have suggested that iron availability and glutathione metabolism are closely interconnected (Geertsema et al., 2026). Although our findings are consistent with this concept, the observational nature of our study precludes conclusions regarding the direction or causality of this relationship. Traditionally, redox alterations in IBD have been interpreted primarily in the context of inflammation-driven oxidative stress (Bourgonje et al., 2019; Geertsema et al., 2026). This distinction may be clinically relevant, as changes in glutathione during therapy could reflect shifts in iron metabolism rather than residual inflammatory activity alone.

This study has several strengths. Most notably, the longitudinal design allowed assessment of both baseline redox status and dynamic changes during induction therapy. A diverse panel of redox biomarkers were measured under standardized conditions, enhancing reliability and reducing confounding. Our inclusion of multiple thiol species and proteins enabled direct comparisons of their relative diagnostic utility for disease activity. Moreover, the combined evaluation of multiple redox biomarkers alongside iron parameters provides a comprehensive view of systemic redox biology. Several limitations should also be acknowledged. The sample size was modest, limiting statistical power, particularly in subgroup analyses, and may explain why some biologically plausible associations did not reach statistical significance. Consequently, these findings should be considered exploratory and hypothesis-generating, and larger prospective studies are needed to validate the observed associations. In addition, subgroup distributions were uneven with respect to treatment type in CD and UC. Although this is not optimal, it likely reflects real-world clinical practice, in which certain therapies are preferentially used in specific patient groups. As a result, apparent treatment-specific differences may, at least in part, reflect underlying disease-specific redox profiles rather than treatment effects alone. Furthermore, the observational nature of the study precludes causal inference. Another limitation is that we measured total circulating thiol species rather than the relative proportions of their reduced and oxidized forms (e.g., GSH/GSSG and Cys/CySS). As these redox couples provide a more direct measure of redox status, their assessment may offer additional mechanistic insight in future studies. Finally, redox biomarkers were measured in the systemic circulation which may not fully reflect local mucosal redox processes.

In conclusion, this study suggests that systemic redox biomarkers may capture distinct and biologically meaningful information during induction therapy in IBD that is not reflected by inflammatory markers alone. Baseline differences and response-dependent changes in cysteine and FTs, particularly in IFX-treated patients, were observed in relation to both short-term and long-term response, suggesting possible underlying differences in systemic redox balance. In addition, glutathione showed strong inverse associations with iron indices, highlighting a possible close interaction between intracellular redox regulation and iron availability that became apparent following effective inflammatory control. Together, these findings may suggest that redox biomarkers, particularly glutathione in relation to iron handling and FTs as markers of systemic redox resilience, provide complementary insight into treatment response and disease biology in IBD. Given the exploratory nature of this study, future studies in larger, independent cohorts are warranted to validate these observations and to determine whether integrating redox and iron biomarkers may improve patient stratification and therapeutic decision-making.

## Data Availability

The original contributions presented in the study are included in the article/Supplementary Material, further inquiries can be directed to the corresponding author.
