# Supplementary material for: Associations between redox-related biomarkers and treatment response in patients with inflammatory bowel disease initiating infliximab or vedolizumab
Source: Front Pharmacol. 2026 Oct 14;17:1908844. doi: 10.3389/fphar.2026.1908844 (PMC13713411; doi:10.3389/fphar.2026.1908844)
Supplement: Supplementary file 1 [file Supplementaryfile1.docx]

Supplementary Material

# 1 Supplementary Data

| **Table S1: Changes in inflammation- and redox biomarkers during induction therapy with either IFX or VEDO in patients with CD** | | | | | |
| --- | --- | --- | --- | --- | --- |
| **IFX** | | | | | |
| **Biomarker** | **Baseline**  **(n=37)** | **Week 6**  **(n=37)** | **Week 14**  **(n=37)** | **Δ Baseline to**  **Week 6** | **Δ Baseline to**  **Week 14** |
| Cysteine (µg/mL) | 3,247 (2,837-3,888) | 2,721 (2,378-3,487) | 3,395 (2,664-4,095) | **<0.05** | 0.87 |
| Homocysteine (ng/mL) | 3,476 (2,979-6,018) | 3,245 (2,973-4,892) | 3,497 (3,061-6,475) | 0.10 | 0.87 |
| Free thiols (µmol/L) | 242.50  (209.36-270.14) | 228.41  (216.18-280.19) | - | 0.94 | - |
| Glutathione (µg/mL) | 79.26 (63.78-96.78) | 72.95 (61.26-91.29) | 78.79 (71.37-103.83) | 0.47 | 0.87 |
| Trx1 (ng/mL) | 106.62 (51.02-355.57) | 169.86 (84.08-536.82) | 176.32 (56.74-406.10) | 0.37 | 0.96 |
| CRP (mg/L) | 2.85 (1.52-7.45) | 1.35 (0.30-1.67) | 1.15 (0.30-3.75) | **<0.05** | 0.16 |
| Fecal calprotectin (mg/kg) | 957.50 (527.50-2,348) | - | 93.00 (40.00-120.00) | - | 0.75 |
| **VEDO** | | | | | |
| **Biomarker** | **Baseline**  **(n=21)** | **Week 6**  **(n=21)** | **Week 14**  **(n=21)** | **Δ Baseline to**  **Week 6** | **Δ Baseline to**  **Week 14** |
| Cysteine (µg/mL) | 3,135 (3,038-4,892) | 4,113 (3,625-5,635) | 4,113 (3,852-4,311) | 0.94 | 1.00 |
| Homocysteine (ng/mL) | 3,289 (2,715-3,504) | 3,664 (3,069-3,934) | 3,725 (3,300-4,054) | 0.38 | 0.38 |
| Free thiols (µmol/L) | 275.56  (230.99-282.77) | 223.73  (213.83-280.41) | - | 0.97 | - |
| Glutathione (µg/mL) | 98.50 (82.57-170.86) | 111.06 (86.73-123.61) | 101.60 (95.70-132.24) | 0.88 | 1.00 |
| Trx1 (ng/mL) | 78.14 (75.01-195.33) | 169.73 (93.76-208.77) | 165.31 (62.40-344.83) | 1.00 | 1.00 |
| CRP (mg/L) | 25.00 (1.60-60.00) | 14.00 (6.00-20.00) | 30.00 (1.70-51.00) | 0.88 | 1.00 |
| Fecal calprotectin (mg/kg) | 286.00  (124.00-709.00) | - | 850.00 (105.00-2,310) | - | 1.00 |
| *Data is presented as median [interquartile range]. Two-tailed P values <0.05 were considered statistically significant; the Benjamini-Hochberg procedure was used to adjust for multiple testing, adopting a 5% false discovery rate. Abbreviations: CRP, C-reactive protein; IBD, inflammatory bowel disease; IFX, infliximab; Trx1, Thioredoxin-1; VEDO, vedolizumab.* | | | | | |

| **Table S2: Changes in inflammation- and redox biomarkers during induction therapy with either IFX or VEDO in patients with UC** | | | | | |
| --- | --- | --- | --- | --- | --- |
| **IFX** | | | | | |
| **Biomarker** | **Baseline**  **(n=37)** | **Week 6**  **(n=37)** | **Week 14**  **(n=37)** | **Δ Baseline to**  **Week 6** | **Δ Baseline to**  **Week 14** |
| Cysteine (µg/mL) | 3,653 (2,831-4,378) | 3,244 (2,468-3,731) | 3,249 (2,316-3,603) | 0.09 | 0.44 |
| Homocysteine (ng/mL) | 3,154 (2,914-3,993) | 3,434 (2,924-4,825) | 3,619 (2,946-4,940) | 0.92 | 1.00 |
| Free thiols (µmol/L) | 212.39  (188.58-232.29) | 259.66  (192.28-303.70) | - | 0.57 | - |
| Glutathione (µg/mL) | 87.58 (81.73-106.97) | 81.85 (68.17-103.96) | 81.92 (55.98-108.80) | 0.37 | 0.55 |
| Trx1 (ng/mL) | 198.27  (115.81-263.14) | 86.08 (61.06-229.31) | 56.13 (41.54-182.98) | 0.57 | 0.30 |
| CRP (mg/L) | 4.50 (1.45-13.50) | 0.60 (0.50-3.60) | 0.80 (0.50-1.20) | 0.09 | 0.14 |
| Fecal calprotectin (mg/kg) | 2,130 (2,075-2,195) | - | 492.50  (396.25-588.75) | - | 1.00 |
| **VEDO** | | | | | |
| **Biomarker** | **Baseline**  **(n=21)** | **Week 6**  **(n=21)** | **Week 14**  **(n=21)** | **Δ Baseline to**  **Week 6** | **Δ Baseline to**  **Week 14** |
| Cysteine (µg/mL) | 3,067 (2,544-4,107) | 3,081 (2,800-3,857) | 3,153 (2,640-3,643) | 0.86 | 0.98 |
| Homocysteine (ng/mL) | 4,207 (2,942-7,362) | 4,161 (3,129-7,866) | 3,858 (3,164-8,601) | 0.52 | 0.73 |
| Free thiols (µmol/L) | 235.95  (204.80-250.53) | 233.12  (222.05-271.77) | - | 0.40 | - |
| Glutathione (µg/mL) | 60.45 (52.77-85.38) | 72.31 (54.39-82.28) | 71.62 (58.00-80.09) | 0.86 | 0.98 |
| Trx1 (ng/mL) | 101.82 (30.00-208.32) | 119.39 (29.23-677.90) | 124.03 (53.30-176.30) | 0.40 | 0.68 |
| CRP (mg/L) | 2.55 (1.18-4.85) | 1.35 (1.05-3.50) | 1.50 (0.73-4.53) | 0.40 | 0.32 |
| Fecal calprotectin (mg/kg) | 650.00 (372.50-1,364) | - | 99.00 (41.00-170.00) | - | **<0.05** |
| *Data is presented as median [interquartile range]. Two-tailed P values <0.05 were considered statistically significant; the Benjamini-Hochberg procedure was used to adjust for multiple testing, adopting a 5% false discovery rate. Abbreviations: CRP, C-reactive protein; IBD, inflammatory bowel disease; IFX, infliximab; Trx1, Thioredoxin-1; VEDO, vedolizumab.* | | | | | |

|  | **Table S3: Plasma biomarker levels and the chance of responding to induction therapy with IFX or biological usage after a year** | | | | |
| --- | --- | --- | --- | --- | --- |
|  |  | **Response after 14 weeks** | | **Biological usage after 1 year** | |
|  |  | **OR per SD of biomarker** | | **OR per SD of biomarker** | |
|  | **Cysteine (baseline)** | | |  | |
|  | Model 1 | 11.23 [0.87-144.68], p=0.06 | | 0.75 [0.32-1.72], p=0.49 | |
|  | Model 2 | No sufficient data | | 1.41 [0.46-4.30], p=0.55 | |
|  | Model 3 | No sufficient data | | 1.63 [0.32-8.35], p=0.56 | |
|  | **Homocysteine (baseline)** | | |  | |
|  | Model 1 | 0.42 [0.16-1.11], p=0.08 | | 0.66 [0.31-1.43], p=0.29 | |
|  | Model 2 | 0.27 [0.00-18.67], p=0.55 | | 0.49 [0.15-1.55], p=0.22 | |
|  | Model 3 | No sufficient data | | 42.58 [0.08-22987.34], p=0.24 | |
|  | **Free thiols (baseline)** | |  |  |  |
|  | Model 1 | 1.35 [0.38-4.78], p=0.64 |  | 0.70 [0.29-1.66], p=0.42 | |
|  | Model 2 | 7.92 [0.11-556.56], p=0.34 |  | 0.49 [0.14-1.68], p=0.25 | |
|  | Model 3 | No sufficient data |  | 0.58 [0.16-2.15], p=0.42 | |
| **Glutathione (baseline)** | | |  |  |  |
| Model 1 | | 0.93 [0.29-2.97], p=0.91 |  | 1.34 [0.49-3.66], p=0.57 | |
| Model 2 | | No sufficient data |  | 1.29 [0.38-4.38], p=0.68 | |
| Model 3 | | No sufficient data |  | 2.06 [0.29-14.63], p=0.47 | |
| **Thioredoxin-1 (baseline)** | | |  |  |  |
| Model 1 | | 16.29 [0.05-5284], p=0.34 |  | 0.81 [0.38-1.72], p=0.59 | |
| Model 2 | | No sufficient data |  | 0.88 [0.27-2.87], p=0.83 | |
| Model 3 | | No sufficient data |  | 0.54 [0.11-2.63], p=0.45 | |
|  | Odds ratios with 95%-confidence intervals obtained from binary logistic regression models. Odds ratios are expressed per 1 standard deviation. Univariable models are without any correction for confounders. Model 1 is crude (unadjusted); Model 2 is Model 1 additionally adjusted for age, sex, BMI and smoking status; Model 3 is Model 2 with further adjustment for biological naïve and immunomodulators usage. P-values <0.05 were considered statistically significant. Abbreviations: OR, odds ratio; SD, standard deviation; 95%CI, 95% confidence interval. | | | | |

| **Table S4: Plasma biomarker levels and the chance of responding to induction therapy with VEDO or biological usage after a year** | | | | | | | | |  |
| --- | --- | --- | --- | --- | --- | --- | --- | --- | --- |
|  | **Response after 14 weeks** | | | | **Biological usage after 1 year** | | | |  |
|  | **OR per SD of biomarker** | | | | **OR per SD of biomarker** | | | |  |
| **Cysteine (baseline)** | | | | |  | | | |  |
| Model 1 | 1.10 [0.41-2.94], p=0.85 | | | | 1.21 [0.43-3.42], p=0.72 | | | |  |
| Model 2 | No sufficient data | | | | No sufficient data | | | |  |
| Model 3 | No sufficient data | | | | No sufficient data | | | |  |
| **Homocysteine (baseline)** | | | | |  | | | |  |
| Model 1 | 3.25 [0.64-16.59], p=0.16 | | | | 2.04 [0.59-7.01], p=0.26 | | | |  |
| Model 2 | No sufficient data | | | | No sufficient data | | | |  |
| Model 3 | No sufficient data | | | | No sufficient data | | | |  |
| **Free thiols (baseline)** | | |  | |  | |  | |  |
| Model 1 | 1.44 [0.54-3.85], p=0.46 | |  | | 2.15 [0.67-6.95], p=0.20 | | | |  |
| Model 2 | 0.95 [0.18-5.09], p=0.96 | |  | | No sufficient data | | | |  |
| Model 3 | No sufficient data | |  | | No sufficient data | | | |  |
| **Glutathione (baseline)** | | | |  | |  | |  | |
| Model 1 | | 0.58 [0.22-1.52], p=0.27 | |  | | 0.42 [0.12-1.53], p=0.19 | | | |
| Model 2 | | No sufficient data | |  | | No sufficient data | | | |
| Model 3 | | No sufficient data | |  | | No sufficient data | | | |
| **Thioredoxin-1 (baseline)** | | | |  | |  | |  | |
| Model 1 | | 0.00 [0.00-5.24], p=0.13 | |  | | 0.00 [0.00-22.82], p=0.21 | | | |
| Model 2 | | 0.23 [0.01-3.94], p=0.31 | |  | | No sufficient data | | | |
| Model 3 | | No sufficient data | |  | | No sufficient data | | | |
| Odds ratios with 95%-confidence intervals obtained from binary logistic regression models. Odds ratios are expressed per 1 standard deviation. Univariable models are without any correction for confounders. Model 1 is crude (unadjusted); Model 2 is Model 1 additionally adjusted for age, sex, BMI and smoking status; Model 3 is Model 2 with further adjustment for biological naïve and immunomodulators usage. P-values <0.05 were considered statistically significant. Abbreviations: OR, odds ratio; SD, standard deviation; 95%CI, 95% confidence interval. | | | | | | | | |  |
